# Supplementary material for: Broad-Spectrum Bactericidal Activity of a Synthetic Random Copolymer Based on 2-Methoxy-6-(4-Vinylbenzyloxy)-Benzylammonium Hydrochloride
Source: Int J Mol Sci. 2021 May 9;22(9):5021. doi: 10.3390/ijms22095021 (PMC8125966; doi:10.3390/ijms22095021)
Supplement: Supplementary file 1 [file ijms-22-05021-s001.zip › ijms-1206859-supplementary.pdf]

Supporting Information

Broad-Spectrum Bactericidal Activity of a Synthetic Random Copolymer based on 2-Methoxy-6-(4-Vinyl benzyloxy)-Benzylammonium Hydrochloride

Anna Maria Schito [1], Gabriella Piatti [1], Debora Caviglia [1], Guendalina Zuccari [2] and Silvana Alfei [2,*]

[1] Department of Surgical Sciences and Integrated Diagnostics (DISC), University of Genoa, Viale Benedetto XV, 6, I-16132 Genova, Italy; amschito@unige.it (A.M.S); gabriella.piatti@unige.it (G.P.), Caviglia86@gmail.com (D.C.)
[2] Department of Pharmacy, University of Genoa, Viale Cembrano, 16148 Genoa, Italy; zuccari@difar.unige.it (G.Z.)
* Correspondence: alfei@difar.unige.it (S.A.); Tel.: +39 010 355 2296

Section S1. Synthesis of 2-Methoxy-6-(4-Vinylbenzyloxy)benzylamonium hydrochloride M7 (**7**) [1].

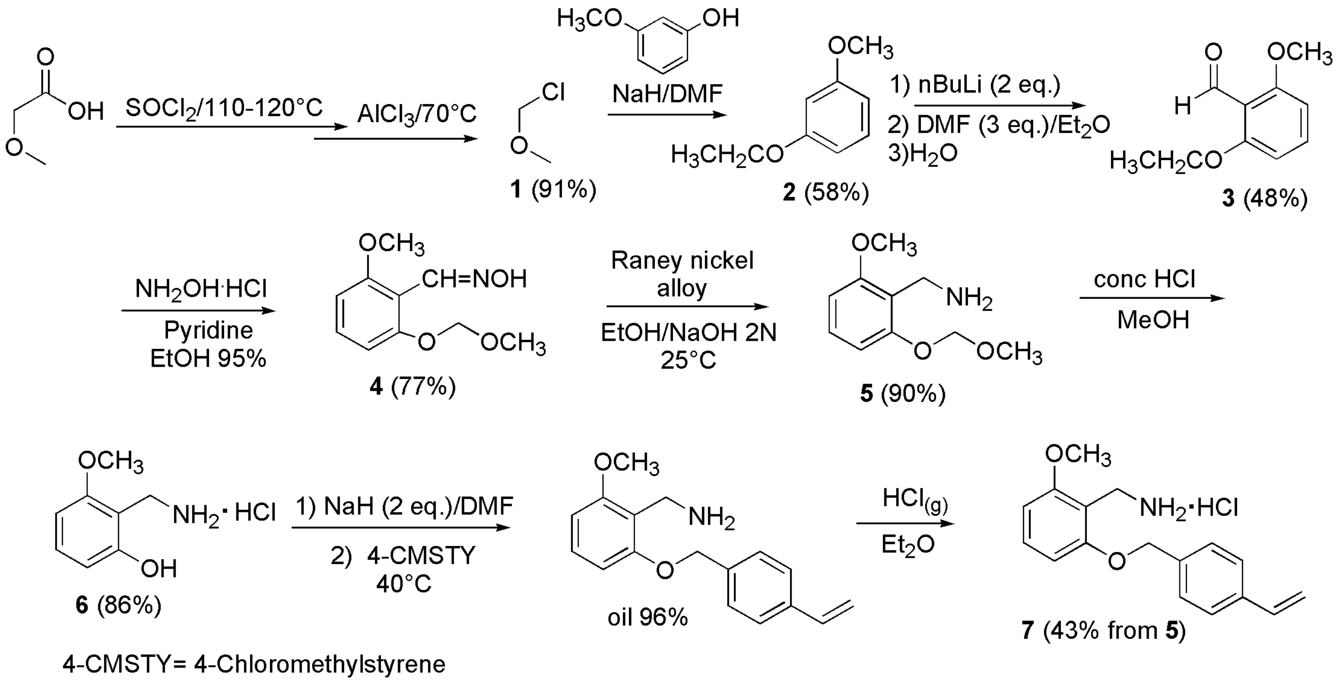

Scheme S1. Synthetic path to prepare M7 (**7**).

*S1.1. Chloromethylmethylether (**1**) [1,2]*

A mixture of methoxyacetic acid (22.50 g, 250.0 mmol) dissolved in freshly distilled thionyl chloride (27.3 mL, 376.0 mmol) was added with a drop of anhydrous dimethylformamide (DMF) and refluxed at 110 °C for 90 'in a single-necked flask with a $CaCl_2$ valve. The stirring was then stopped, and the mixture was allowed to cool at r.t. and added with $AlCl_3$ (0.35 g, 2.60 mmol) causing intense foaming. The new mixture was heated at 70 °C for 30'. At the end, the reaction mixture was transferred into a Claisen instrument equipped with a deflammator arm, filled with glass spheres with an average diameter of 2 mm, and distilled at 1 a.t.m. by heating with a thermostated bath at 160 °C. Three fractions were collected as follows.
Fraction 1. Bp. = 53 °C, 1.05 g, which was discarded.
Fraction 2. Bp. = 59-63 ° C, 6.96 g, compound **1** polluted of the undecomposed intermediate acid chloride.
Fraction 3. Bp. = 72-75 °C, 13.73 g, undecomposed intermediate acid chloride.

Fraction 3 was reacted again with $AlCl_3$ (0.24 g, 1.80 mmol) as previously described and redistilled collecting a single fraction (10.41 g) having b. p. of 57-59 °C.
This fraction was joined to fraction 2 obtained from the first distillation and the collected fractions were redistilled obtaining the pure compound **1** (18.42 g, 229.0 mmol, 91% yield).
Bp. 59-63 °C (Lit. [2] 59-60 °C). IR (film, ν cm^{-1}) 1232, 1119 (C-O-C), 650 (C-Cl).

*S1.2. 3-Methoxymethoxyanysole (**2**) [1,3]*

A 60 % mineral oil dispersion of NaH (4.6 g for actual 2.73 g, 114.0 mmol) was placed in a 250 mL, three-necked, round-bottomed flask equipped with a reflux condenser, a funnel and flushed with N_2, and was washed free of mineral oil with five small portions of pentane. DMF (90 mL) were added and the suspension was stirred by a magnetic bar. The commercial 3-hydroxyanysole (13.98 g, 112.6 mmol) dissolved in 28 mL of dry DMF was added slowly (over 15 min) to the stirred mixture at 0 °C. The reaction was stirred at r.t. for 2 h. The chloromethyl methyl ether (9.97 g, 124.0 mmol) was added slowly and the reaction was followed with TLC and was complete within 3 h after the addition of chloromethyl methyl ether. The reaction mixture was hydrolyzed with NaOH 10% (80 mL, pH = 14) and the aqueous layer was separated and extracted five times with ether (30 mL). The collected ether extracts were dried overnight on $MgSO_4$. After removal of the solvent at reduced pressure, the crude oily product was purified by distillation under vacuum to yield compound **2** with a good degree of pureness (10.92 g, 64.9 mmol, 57.6 % yield). Bp. 111 °C (17 mm); Purity 95 % by HPLC, 94% by GC; IR (film, ν cm^{-1}) 2836 (MeO), 1593, 1492 (C=C, phenyl), 1146, 1014 (C-O-C). ^1H NMR (CCl_4, 300 MHz, δ ppm) 3.44 (s, 3H), 3.74 (s, 3H), 5.13 (s, 2H), 6.2-7.5 (m, 4H). Anal. Calcd. for $C_9H_{12}O_3$: C, 64.27; H, 7.19. Found: C, 64.57; H, 7.43. GC/MS: 168 (M^+, 100%); 138 ($C_8H_{10}O_2^+$, 13%); 45 ($C_2H_5O^+$, 36%).

*S1.3. 2-Methoxy-6-methoxymethoxybenzaldehyde (**3**) [1,4]*

3-methoxymethoxyanysole (16.33 g, 97.1 mmol) was dissolved in dry Et_2O (180 mL) and the obtained solution was placed in a 500 mL, three-necked, round-bottomed flask equipped with a reflux condenser and a funnel and were added slowly (over 15 min) with a freshly titrated 1.64 N butyllithium solution. The mixture was stirred at r.t. for 90′ with formation of precipitate. Dry DMF (24.3 mL) dissolved in dry Et_2O (36 mL) was added and the suspension was stirred for additional 30′ and hydrolyzed with water (110 mL). The organic phase was separated, and the alkaline water was extracted four times with ether (30 mL). The collected ether extracts were dried overnight on $MgSO_4$. After removal of the solvent at reduced pressure, the crude oily product was purified by column chromatography (eluent benzene/ethyl acetate 60/40) to achieve **3** (8.97 g, 45.6 mmol, 48 % yield). IR (film, ν cm^{-1}) 2839 (MeO), 1690 (C=O), 1597, 1475 (C=C, phenyl), 1156, 1076 (C-O-C). ^1H NMR ($CDCl_3$, 300 MHz, δ ppm) 3.51 (s, 3H), 3.90 (s, 3H), 5.25 (s, 2H), 6.66 (d, 1H, J_o = 8.4 Hz), 6.79 (t, 1H, J_o = 8.4 Hz), 7.46 (d, 1H, J_o = 8.4 Hz), 10.51 (s, 1H). ^{13}C NMR (75.5 MHz, δ ppm) 55.88, 56.31, 95.00, 105.00, 107.19, 115.44, 135.87, 160.00, 161.75, 189.13. GC/MS: 196 (M^+, 27%); 150 ($C_8H_8O_2^+$, 61%); 45 ($C_2H_5O^+$, 36%).

*S1.4. 2-methoxy-6-methoxymethoxybenzaldoxime (**4**)*

A solution of **3** (1.08 g; 5.49 mmol) in 95% ethanol (13 mL) was treated with a solution of hydroxylamine hydrochloride (0.46 g, 6.59 mmol) in dry pyridine (2.2 mL) under stirring at room temperature for 1h 20 min and at 0 °C for 30 min to facilitate the oxime precipitation. The white solid was filtered, dried, weighed (0.82 g, 3.89 mmol). The mother liquors were concentrated to afford additional 0.0435 g of 2-methoxy-6-methoxymethoxybenzaldoxime (**4**) for an overall yield of 75 %. Mp 131–135 °C; FTIR (KBr, ν cm^{-1}) 3195 (OH), 2836 (MeO); 1627 (C=N), 1593, 1476 (C=C phenyl), 1072, 1000 (C-O-C).

*S1.5. 2-methoxy-6-methoxymethoxybenzylamine (**5**)*

A solution of **4** (3.13 g; 14.8 mmol) in 95% ethanol (43 mL) was treated with an equal volume of 2 M NaOH followed by Raney nickel alloy (4.67 g) under stirring at room temperature for 90 min. The Raney nickel alloy was removed by filtration and washed with fresh ethanol. Filtrate and washings were combined, acidified with 0.8 M HCl (230 ml) and extracted with CH_2Cl_2 (30 mL). The aqueous phase was treated with solid KOH up to pH = 14 and extracted with diethyl ether (3x30 mL). The extracts after drying over anhydrous $MgSO_4$ and removal of the solvent afforded 2-methoxy-6-methoxymethoxybenzylamine (**5**) (2.64 g, 13.40 mmol, 90 %

yield). Bp 70 °C/0.02 torr. Purity 98% by GC-FID. FTIR (KBr, ν cm^{-1}) 3409, 3340 (NH), 2848 (MeO), 1594, 1472 (C=C phenyl), 1152, 1075 (C-O-C). ^{1}H NMR ($CDCl_3$, 300 MHz, δ ppm) 1.61 (bs, 2H); 3.48 (s, 3H); 3.82 (s, 3H); 3.89 (s, 2H); 5.20 (s, 2H); 6.58 (d, 1H, Jo = 8.0 Hz); 6.73 (d, 1H, Jo = 8.0 Hz); 7.14 (t, 1H, Jo = 8.0 Hz).

*S1.6. 2-hydroxy-6-methoxymethoxybenzylamine hydrochloride (**6**)*

A mixture of **5** (2.93 g, 14.9 mmol), methanol (150 mL) and hydrochloric acid (5 mL) was heated at 62 °C under stirring for 20 min up to the disappearance of **5** (TLC, eluent benzene/ethyl acetate = 90/10). After removal of the solvent at reduced pressure the solid residue was dissolved in the minimum amount of DMF and precipitated in chloroform to afford **6** in the form of pearly flakes (2.03 g, 10.71). The mother liquor after concentration and cooling afforded additional **6** (0.39 g, 2.04 mmol), overall yield 86%. Mp 211–214 °C. FTIR (KBr, ν cm^{-1}) 3427 (OH) 3197, 3026 (NH_3^+), 2837 (MeO), 1603 (NH), 1573, 1503, 1473 (C=C phenyl), 1120 (C-O-C). ^{1}H NMR (CD_3OD, 300 MHz, δ ppm) 3.86 (s, 3H); 4.16 (s, 2H); 4.88 (s, 4H); 6.54 (m, 2H); 7.19 (m, 1H). ^{13}C NMR (75.5 MHz, δ ppm) 33.66, 56.30, 103.12, 108.60, 109.21, 131.99, 158.21, 160.33.

*S1.7. 2-Methoxy-6-[(4-vinyl)-benzyloxy]-benzylammonium hydrochloride M7 (**7**)*

Sodium hydride (1.05 g, 25.3 mmol) as a 60% dispersion in mineral oil was washed three times with pentane under nitrogen and suspended in dry DMF (123 mL). The suspension was added with a solution of **6** (2.49 g, 13.11 mmol) in dry DMF (10.5 mL), stirred for 90 min and treated with commercial chlomethylstyrene (1.9 mL, 15.11 mmol) under stirring at 40 °C for 3 h. The reaction mixture was hydrolyzed with 10% aqueous NaOH (40 mL, pH = 14) and extracted with peroxide-free diethyl ether (3 x 30 mL). The extracts after drying over anhydrous $MgSO_4$ and removal of the last traces of DMF under vacuum afforded the free amine as crude oil (3.38 g, 12.00 mmol, 95.8 % yield). Without further purification, 3.24 g (12.01 mmol) of free oily amine were soon converted into its hydrochloride **7** by dissolution in dry diethyl ether (200 mL). The clear solution was cooled to 0 °C and treated under stirring up to saturation with dry gaseous hydrochloric acid (30′). The hydrochloride **7**, precipitated as white flaky solid (3.61 g) was filtered, washed several times with fresh anhydrous ether, dried and recrystallized from ACN to obtain a white crystalline solid (1.07 g, 3.50 mmol). The mother liquors placed in the refrigerator provided an additional 0.43 g of **7** for an overall yield of 43%. Mp 215–218 °C (acetonitrile). Purity 99% by HPLC. FTIR (KBr, ν cm^{-1}) 3500-3000 (NH_3^+); 1599, 1474 (C=C, phenyl) 999 and 923 (CH_2=CH). ^{1}H NMR (CD_3OD, 300 MHz, δ ppm) 3.90 (s, 3H), 4.20 (s, 2H); 5.16 (s, 2H); 5.23 (dd, 1H, J_{gem} = 1.0 Hz; J_{cis} = 10.9 Hz); 5.82 (dd, 1H, J_{gem} = 1.0 Hz; J_{trans} = 17.6 Hz); 6.70–6.80 (m, 3H); 7.33–7.45 (m, 5H). ^{13}C NMR (75.5 MHz, δ ppm) 33.41, 56.45, 71.50, 105.06, 106.50, 110.26, 114.46, 127.43, 128.95, 132.46, 137.70, 137.73, 138.95, 159.05, 160.28. Anal. Calcd. for $C_{17}H_{20}ClNO_2$: C, 66.77; H, 6.59; N, 4.58; Cl, 11.59. Found: C, 66.80; H, 6.58; N, 4.60; Cl, 11.62.

Section S2. Preparation and Characterization of Copolymer P7 by Radical Copolymerization in Solution [1].

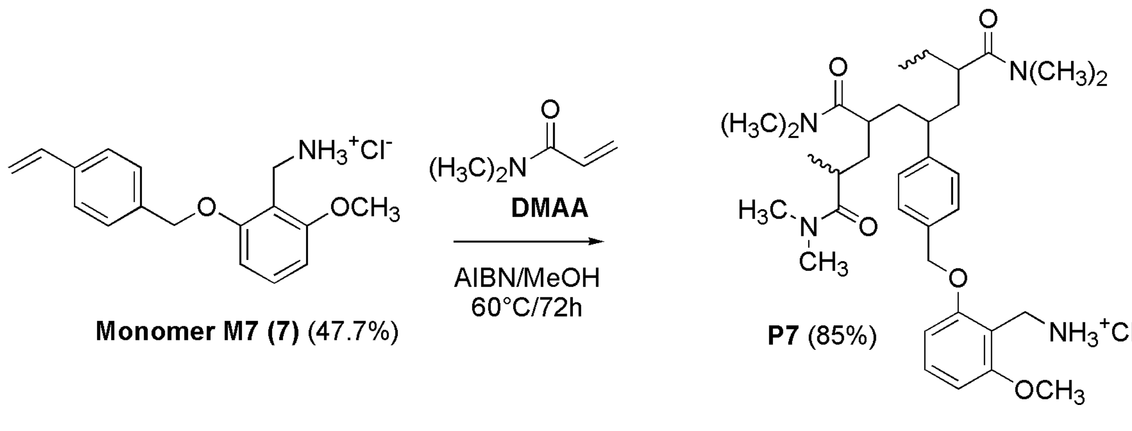

Scheme S2. Reaction scheme of copolymerization of M7 with DMAA to effort P7.

The experimental data of the copolymerization have been reported in the following Table S1.

Table S1. Experimental data of copolymerization of M7 with DMAA.

M7 (mg, mmol, %[1])	DMAA (mg, mmol)	MeOH (mL)	AIBN (mg, %[2])	Time (h)	P7 (g, %[3])
734.9, 2.68, 47.7	560.0, 5.63	MeOH 19	13.4, 1.7	72	1.10, 85

[1] loading (%) of cationic monomer; [2] percentage wt/wt (A+B); [3] percentage of conversion.

S2.1. Synthetic Procedure

In a 25 mL tailed test tube equipped with a magnetic stirrer and carefully flamed under nitrogen, M7 (**7**), DMAA, AIBN as radical initiator, and freshly distilled anhydrous MeOH were introduced in the ratios reported in Table S1. The mixture thus obtained were subjected to three vacuum-nitrogen cycles to remove the oxygen. The clear solution was then siphoned into a 25 mL flask with screw cap and silicone septum. Nitrogen was then bubbled for 3 minutes in the solution, which was subsequently left under stirring at 60 °C. The final yellow solution was evaporated at reduced pressure achieving the crude co-polymer, which was subjected to three cycles of dissolution in MeOH and precipitation in Et_2O obtaining P7 as white solid. P7 was subsequently subjected to fractioning as described in the following Section S2.1.1.

S2.1.1. Fractioning of P7

A solution of P7 in just enough MeOH was filtered and transferred in a three-necks round-bottomed flask equipped with a mechanic stirrer and a funnel. It was thermostated at 25 ° C and the clear solution (S1) was slowly added with Et_2O until a milky precipitate (MP7-1) was obtained. MP7-1 was separated from the supernatant (S2) by centrifugation at 3000 rpm for 15′.

S2 was treated as the starting solution (S1) obtaining a second milky precipitate (MP7-2). MP7-1 and MP7-2 were then dissolved in MeOH and precipitated in an excess of Et_2O obtaining the corresponding copolymers, namely P7-High and P7-Low.

FTIR (KBr, ν cm^{-1}) 3500 (NH_3^+); 2800-2900 (C-H stretching alkyl groups); 2000-1700 (aromatic overtones); 1649 (C=ONH); 1575 and 1510 (aromatic -C=C- stretching); 754 (*o*-disubstituted phenyl ring).

The unreacted monomer M7 (**7**) was recovered from the mixture of the combined solvents by evaporation at reduced pressure.

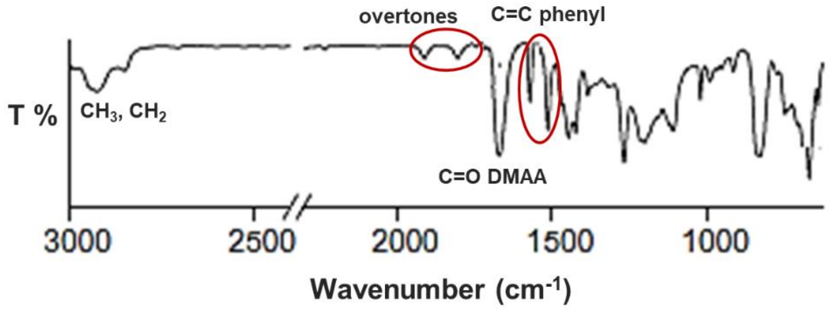

Figure S1. Significant part of FTIR spectrum of P7 (bands over 3000 cm^{-1} omitted).

S2.2. Determination of the Average Molecular Mass (Mn) of Copolymer P7

S2.2.1. Calibration Phase

Solutions of polyoxyethylene (PEO) with Mn 10,800 in MeOH were prepared at three different concentrations [c (mol/Kg)] and were analyzed by the vapor pressure osmometer (VPO) technique at 45 °C. The quotients of measured values (MV) and the corresponding concentrations, i. e. MV/c (Kg/mol), were determined. From these data (Table S2), by using the Ordinary Least Squares (OLS) method, a linear regression curve was obtained, whose equation was Eq. (1).

$$y = 73442x + 500.92 \quad (1)$$

Extrapolating it to concentration c = 0, K_{cal} was determined and was found to be 501.

S2.2.2. Measurements Phase

Solutions of copolymer P7 in MeOH were prepared at three different concentrations c (g/Kg) (Table S2) and were analyzed by VPO method at 45 °C. The ratios between the measurement values (MV) and concentrations (c) (kg/g) were plotted *vs* concentrations (c) and by using the OLS method, a linear regression curve was obtained, whose extrapolation to concentration c = 0 provided the K_{meas} (kg/g) value for P7 (Table S2). The average molecular mass (Mn) of P7 was determined with equation Eq. (2) and was reported in Table S2.

$$MW\left(\frac{g}{mol}\right) = \frac{Kcal}{Kmeas} \quad (2)$$

Table S2. Data of calibration, results of measurements and estimation of Mn of P7 according to Eq. (2).

**Calibration**		**Measurements**	
**c (mol/Kg)^{PEO}**	**MV/c (Kg/mol)^{PEO}**	**c (g/Kg)^{P7}**	**Mn (g/mol)**
0.0048410	856	13.8525	
0.0058304	930	23.9796	13,719
0.0068966	1007	30.5577	
**K_{cal} (Kg/mol)** = 501		**K_{meas} (Kg/g)** = 0.0365	

S2.3. Determination of NH_2 Equivalents contained in P7

The NH_2 equivalents, in the form of hydrochloride salts contained in P7, were determined by volumetric titrations with a solution of $HClO_4$ in acetic acid (AcOH), using quinaldine red as indicator [5]. Briefly, acetic anhydride (3 mL) was added to a solution of $HClO_4$ 70% (1.4 mL) in AcOH (80 mL), obtaining a colorless solution which was left under stirring at room temperature overnight. The clear yellow solution was made up to 100 mL with AcOH and standardized with potassium acid phthalate. The title of solution was found to be 0.1612 N. A sample of P7 (350.1 mg) was dissolved in AcOH (5 mL), treated with 2 mL of a solution of mercury acetate (1.5 g) in AcOH (25 mL), added with a few drops of a solution of quinaldine red (100 mg) in AcOH (25 mL) and titrated with the standardized solution of $HClO_4$ in AcOH, using a calibrated burette with needle valve (0.02 mL). The very sharp end points were detected by observing the disappearance of the red color. Standardization and titrations were made in triplicate and the results were reported as means ± standard deviation (SD). The content of NH_2 was expressed both as μequiv. of NH_2 per μmol of P7 and as μequiv. of NH_2 per gram of P7 and reported in Table S3.

Table S3. Results of volumetric titration of an exactly weighted amount of P7.

**P7 (13,719)[1] mg (mmol)**	**$HClO_4$ 0.1612 N (mL)**	**NH_2 (mmol)**	**$\mu mol_{NH2}/g_{P7}$**	**$\mu mol_{NH2}/\mu mol_{P7}$**
350.1 (0.0255)	0.66	0.1067	305	4.2

[1] Mn of P7.

S2.4. Potentiometric Titration of P7

The herein tested copolymer P7 had not quaternary and permanently protonated ammonium groups but was characterized by having reversibly protonable primary amine groups, depending on pH value of the environment. The protonation of amine groups of nanomaterials, intended to work as an antibacterial or chemotherapeutic agent, is essential for having a positively charged surface, crucial to provide significant cytotoxic activity. Therefore, to suggest a possible clinical application of P7, it was important to know the pH values at which it can be protonated and mainly, if P7 will be protonated in the physiological pH range of 4.5-

7.5. To obtain this information, the potentiometric titration of P7 has been carried out according to Benns et al. [6] and the data has been reported in Table S4.

Briefly, potentiometric titrations were performed at room temperature to construct the titration curve of P7. The copolymer (30 mg) was dissolved in 30 mL of Milli-Q water (mQ), then was treated with a standard 0.1 N NaOH aqueous solution [1.5 mL, pH = 9.54]. The solution was potentiometrically titrated by adding 0.2 mL aliquots of a standard 0.1 N HCl aqueous solution, up to total 3.0 mL and measuring the corresponding pH values [6]. For comparison purposes commercial branched polyethyleneimine (PEI-*b*) 25kD was titrated in the same conditions.

Table S4. Data of potentiometric titration of P7 used to construct the titration curve (Figure S2, red line) and computed values of dpH/dV used to construct the relative first derivative curve (Figure S, purple line). The last three rows report the max values of dpH/dV computed for P7 and the corresponding values of HCL 0.1 N volumes and of pH.

mL HCl 0.1N*	pH(P7)*	dpH/dV(P7)
0.0	9.54	
0.2	9.30	1.2
0.4	9.00	1.5
0.6	6.85	10.75
0.8	6.15	3.5
1.0	5.60	2.75
1.2	4.80	4
1.4	4.65	0.75
1.6	4.50	0.75
1.8	4.45	0.25
2.0	4.40	0.25
2.2	4.35	0.25
2.4	4.30	025
2.6	4.30	0
2.8	4.20	0.5
3.0	4.15	0.25
Max dpH/dV	4.5	4
HCl (mL)	1.2	1.2
pH	5.20	4.80

*The results of potentiometric titration were reported as means of three replicates.

By reporting in graph, the measured pH values versus the aliquots of HCl 0.1 N added, the titration curve of P7 was obtained (Figure S2, red line). Subsequently, from titration data, the dpH/dV values were computed and reported in Table S4. By reporting in graph these values versus those of the corresponding volumes of HCl 0.1N, the first derivative line of the titration curve was obtained (Figure S2, purple line). The maxima of these latter curve corresponded to the volumes of HCl necessary to have P7 in the protonated form. Interestingly, two maxima were observed, thus establishing for the existence of a two-step protonation process. Data reported in last three rows of Table S4, proved that P7 in the physiological pH range should be completely protonated, as desired.

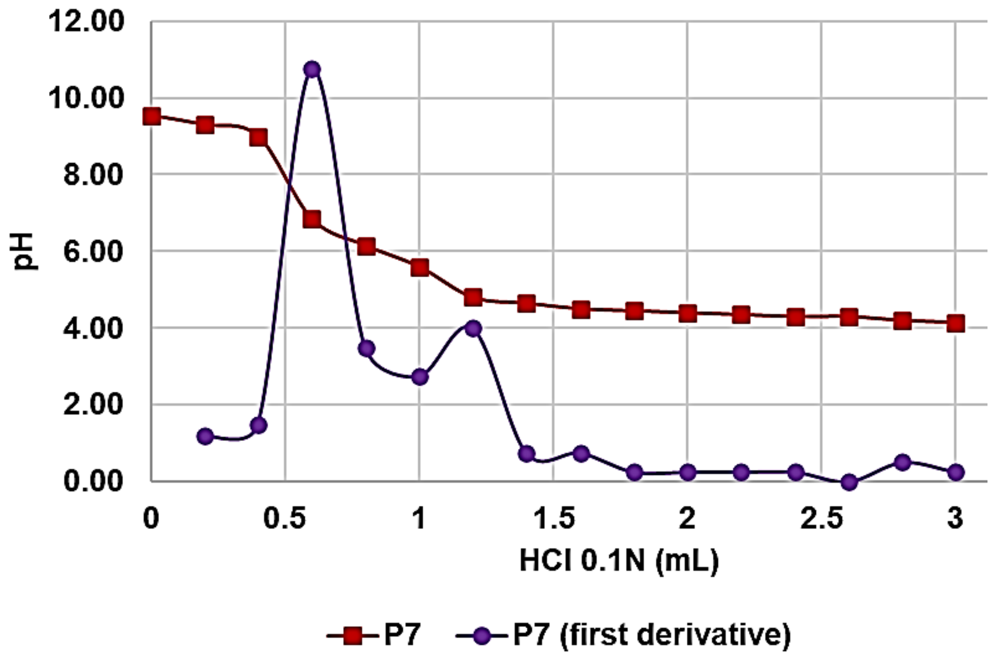

Figure S2. Titration curve of P7 (**red line**); first derivatives lines of the titration curve (**purple line**).

S2.5. Z-potential (ζ-p) and Dynamic Light Scattering (DLS) Analysis of P7

ζ-p and dynamic light scattering (DLS) measurements of P7 were performed in aqueous solutions (mQ) (pH = 7.4) at a fixed concentration of 1 mg/mL and at the physiological temperature of 37°C. The excitation light source was a 4 mW He–Ne laser at 633 nm and the intensity of the scattering angle was fixed at 173 °C. Results were the combination of three 10-min runs for a total accumulation correlation function (ACF) time of 30 min. From the relaxation time determined, it was estimated the apparent diffusion coefficient, D, at the actual co-polymer concentration. The apparent hydrodynamic radius (R_{hyd}), of the particles was calculated through the Stokes–Einstein equation [7] and was reported as the mean of three measurements ± SD.

PDI value was reported as the mean of three measurements ± SD made by the instrument on the sample. The ζ-p was measured at 37° C in mQ water as a medium, and an applied voltage of 100 mV was used. The P7 sample was loaded into pre-rinsed folded capillary cells, and twelve measurements were performed (Table S5).

Table S5. Hydrodynamic size (nm), Z-potential and PDI at 37 °C of P7 by DLS analysis (degree of freedom N = 3).

Copolymer	Hydrodynamic diameter (nm)	PDI	Z-Potential (mV)
P7	220 ± 18	0.809 ± 0.004	+49.8 ± 5.8

References

1. Alfei, S.; Marengo, B.; Valenti, G.E.; Domenicotti, C. Synthesis of Polystyrene-Based Cationic Nanomaterials with Pro-Oxidant Cytotoxic Activity on Etoposide-Resistant Neuroblastoma Cells. *Nanomaterials* **2021**, *11*, 977. https://doi.org/10.3390/nano11040977.
2. Stadlwieser, J. Notiz zur Herstellung von Chloromethylmethylether aus Methoxyessigsäure. *Sytnthesis* **1985**, *5*, 490.

3. Winkle, M.R.; Ronald, R.C. Regioselective metalation reactions of some substituted (methoxymethoxy)arenes. *J. Org. Chem.* **1982**, *47*, 2101–2108, doi:10.1021/jo00132a022.
4. Narasimhan, N.S.; Mali, R.S.; Barve, M.V., *Synthesis* **1979**, *11*, 906.
5. Alfei, S.; Castellaro, S. Synthesis and Characterization of Polyester-Based Dendrimers Containing Peripheral Arginine or Mixed Amino Acids as Potential Vectors for Gene and Drug Delivery. *Macromol. Res.* **2017**, *25*, 1172–1186.
6. Benns, J. M.; Choi, J. S.; Mahato, R. I.; Park, J. S.; Kim, S. W. pH-sensitive cationic polymer gene delivery vehicle: N-Ac-poly(L-histidine)-graft-poly(L-lysine) comb shaped polymer. *Bioconj. Chem.* **2000**, *11*, 637-645.
7. Koromilas, N.D.; Lainioti, G.Ch.; Oikonomou, E.K.; Joannis, G.B.; Kallitsis, K. Synthesis and self-association in dilute aqueous solution of hydrophobically modified polycations and polyampholytes based on 4-vinylbenzyl chloride. *Eur. Pol. J.* **2014**, *54*, 39–51.
